# New Surveillance Metrics for Alerting Community-Acquired Outbreaks of Emerging SARS-CoV-2 Variants Using Imported Case Data: Bayesian Markov Chain Monte Carlo Approach

**DOI:** 10.2196/40866

**Published:** 2022-11-25

**Authors:** Amy Ming-Fang Yen, Tony Hsiu-Hsi Chen, Wei-Jung Chang, Ting-Yu Lin, Grace Hsiao-Hsuan Jen, Chen-Yang Hsu, Sen-Te Wang, Huong Dang, Sam Li-Sheng Chen

**Affiliations:** 1 School of Oral Hygiene College of Oral Medicine Taipei Medical University Taipei Taiwan; 2 Institute of Epidemiology and Preventive Medicine College of Public Health National Taiwan University Taipei Taiwan; 3 Daichung Hospital Miaoli Taiwan; 4 Department of Family Medicine Taipei Medical University Hospital Taipei Taiwan; 5 Department of Family Medicine School of Medicine, College of Medicine Taipei Medical University Taipei Taiwan; 6 Department of Economics and Finance University of Canterbury Christchurch New Zealand; 7 Research Center of Cancer Translational Medicine Taipei Medical University Taipei Taiwan

**Keywords:** COVID-19, imported case, surveillance metric, early detection, community-acquired outbreak

## Abstract

**Background:**

Global transmission from imported cases to domestic cluster infections is often the origin of local community-acquired outbreaks when facing emerging SARS-CoV-2 variants.

**Objective:**

We aimed to develop new surveillance metrics for alerting emerging community-acquired outbreaks arising from new strains by monitoring the risk of small domestic cluster infections originating from few imported cases of emerging variants.

**Methods:**

We used Taiwanese COVID-19 weekly data on imported cases, domestic cluster infections, and community-acquired outbreaks. The study period included the D614G strain in February 2020, the Alpha and Delta variants of concern (VOCs) in 2021, and the Omicron BA.1 and BA.2 VOCs in April 2022. The number of cases arising from domestic cluster infection caused by imported cases (Dci/Imc) per week was used as the SARS-CoV-2 strain-dependent surveillance metric for alerting local community-acquired outbreaks. Its upper 95% credible interval was used as the alert threshold for guiding the rapid preparedness of containment measures, including nonpharmaceutical interventions (NPIs), testing, and vaccination. The 2 metrics were estimated by using the Bayesian Monte Carlo Markov Chain method underpinning the directed acyclic graphic diagram constructed by the extra-Poisson (random-effect) regression model. The proposed model was also used to assess the most likely week lag of imported cases prior to the current week of domestic cluster infections.

**Results:**

A 1-week lag of imported cases prior to the current week of domestic cluster infections was considered optimal. Both metrics of Dci/Imc and the alert threshold varied with SARS-CoV-2 variants and available containment measures. The estimates were 9.54% and 12.59%, respectively, for D614G and increased to 14.14% and 25.10%, respectively, for the Alpha VOC when only NPIs and testing were available. The corresponding figures were 10.01% and 13.32% for the Delta VOC, but reduced to 4.29% and 5.19% for the Omicron VOC when NPIs, testing, and vaccination were available. The rapid preparedness of containment measures guided by the estimated metrics accounted for the lack of community-acquired outbreaks during the D614G period, the early Alpha VOC period, the Delta VOC period, and the Omicron VOC period between BA.1 and BA.2. In contrast, community-acquired outbreaks of the Alpha VOC in mid-May 2021, Omicron BA.1 VOC in January 2022, and Omicron BA.2 VOC from April 2022 onwards, were indicative of the failure to prepare containment measures guided by the alert threshold.

**Conclusions:**

We developed new surveillance metrics for estimating the risk of domestic cluster infections with increasing imported cases and its alert threshold for community-acquired infections varying with emerging SARS-CoV-2 strains and the availability of containment measures. The use of new surveillance metrics is important in the rapid preparedness of containment measures for averting large-scale community-acquired outbreaks arising from emerging imported SARS-CoV-2 variants.

## Introduction

During the COVID-19 pandemic lasting for over 2.5 years, countries around the world have experienced cyclical COVID-19 changes alternating between lifting and operating nonpharmaceutical interventions (NPIs) and between the protective and waning effects of vaccines when facing the incessant epidemics of the COVID-19 pandemic [1-4]. The cyclical resurgence of COVID-19 at the country, continental, and global levels is mainly caused by emerging SARS-CoV-2 variants, particularly variants of concern (VOCs). In response to the resurgence of community-acquired outbreaks, 2 containment measures have become important, including the timely adjustment of NPIs (strengthened border control strategies and restricted social activities) combined with testing and the launch of mass primary and booster vaccinations [5-7].

It should be noted that the typical pattern of transmission from an imported case to domestic cluster infection is often the root of local community-acquired outbreaks caused by emerging SARS-CoV-2 variants from any region or country across the globe [2,7-9]. Such an importation-cluster transmission mode has been clearly demonstrated by the resurgence of global epidemic waves following the emergence of dominant strains of Alpha, Beta, Gamma, Delta, and Omicron VOCs. To avert local community-acquired outbreaks of emerging SARS-CoV-2 variants, rapid preparedness of containment measures and effective contact tracing are mandatory when domestic cluster infections are identified after the introduction of emerging imported cases. In addition, the risk of domestic cluster infection on the introduction of imported cases varies with each emerging SARS-CoV-2 strain owing to the evolutionary characteristics of invading VOCs, including an increase in transmissibility and a higher likelihood of escaping immune response after vaccination [4,7-12].

It is therefore important to have new surveillance metrics for monitoring the odds of having domestic cluster infection transmitted from few imported cases and setting up the alert threshold for forestalling community-acquired outbreaks, as traditional surveillance metrics, like effective reproductive number (R_t_), are tailored for assessing the spread and control of community-acquired outbreaks at the population level, which may only involve a single country and a specific SARS-CoV-2 strain in a short period rather than the country of the imported case across the world and the full spectrum of SARS-CoV-2 strains with a long period [13-16]. Such a traditional epidemic surveillance model (eg, the SEIR [Susceptible-​Exposed-Infected-Recovery] model) is not only limited to model the relationship of sparse cases of domestic cluster infection and small samples of imported cases from each original country, but also inflexible to make allowance for the heterogeneity of the imported-domestic transmission mode across countries and SARS-CoV-2 strains across time, as well as the variation across local regions in question. To consider these issues of heterogeneity, it is therefore necessary to develop new surveillance models and their corresponding metrics with a new statistical approach, such as a sampling method of machine learning, particularly the Bayesian Markov Chain Monte Carlo (MCMC) method, in conjunction with a sparse event history regression model, such as the extra-Poisson (random-effect) regression model with relevant parameters and random variables parameterized under the directed acyclic graphic (DAG) diagram.

Developing these new surveillance metrics for quantifying the effect size of the transmission from importation to domestic cluster infection would not only be helpful for alerting emerging community-acquired outbreaks, but also aid health professionals having rapid preparedness of SARS-CoV-2 strain–dependent containment measures, including effective and efficient contact tracing. Using a series of chronological epidemic data on COVID-19 divided into 2 phases (non-VOC phase [wild type and D614G] and VOC phase) in Taiwan, this study aimed to develop new surveillance metrices across the periods of various SARS-CoV-2 strains for alerting emerging community-acquired outbreaks by monitoring the risk of small domestic cluster infections originating from the transmission of few imported cases of emerging variants in order to forestall community-acquired outbreaks when facing emerging SARS-CoV-2 variants. The Bayesian MCMC sampling method was therefore used to estimate and predict the new surveillance metrics underpinning the DAG diagram of the Poisson or negative binomial random-effect regression model.

## Methods

### Data Sources

Publicly available information on COVID-19, including the daily number of cases, recovered patients, and deaths from January 1, 2020, to April 2, 2022, in Taiwan, was extracted from the report of the Central Epidemic Command Centre and the Taiwan National Infectious Disease Statistics System maintained by the Taiwan Centre for Disease Control [17]. During the period between January 11 and June 20, 2020, tabular data with epidemiologic information on COVID-19 mentioned above by county and origin of cases (domestic versus imported) were obtained. After October 2020, only aggregated numbers of imported and domestic COVID-19 cases without detailed information at the county and city levels were provided. The population sizes of 23 counties and cities in Taiwan were extracted from the official website of the Department of Household Registration [18].

### Containment Measures for the Non-VOC Phase in Taiwan

**Figure 1:**
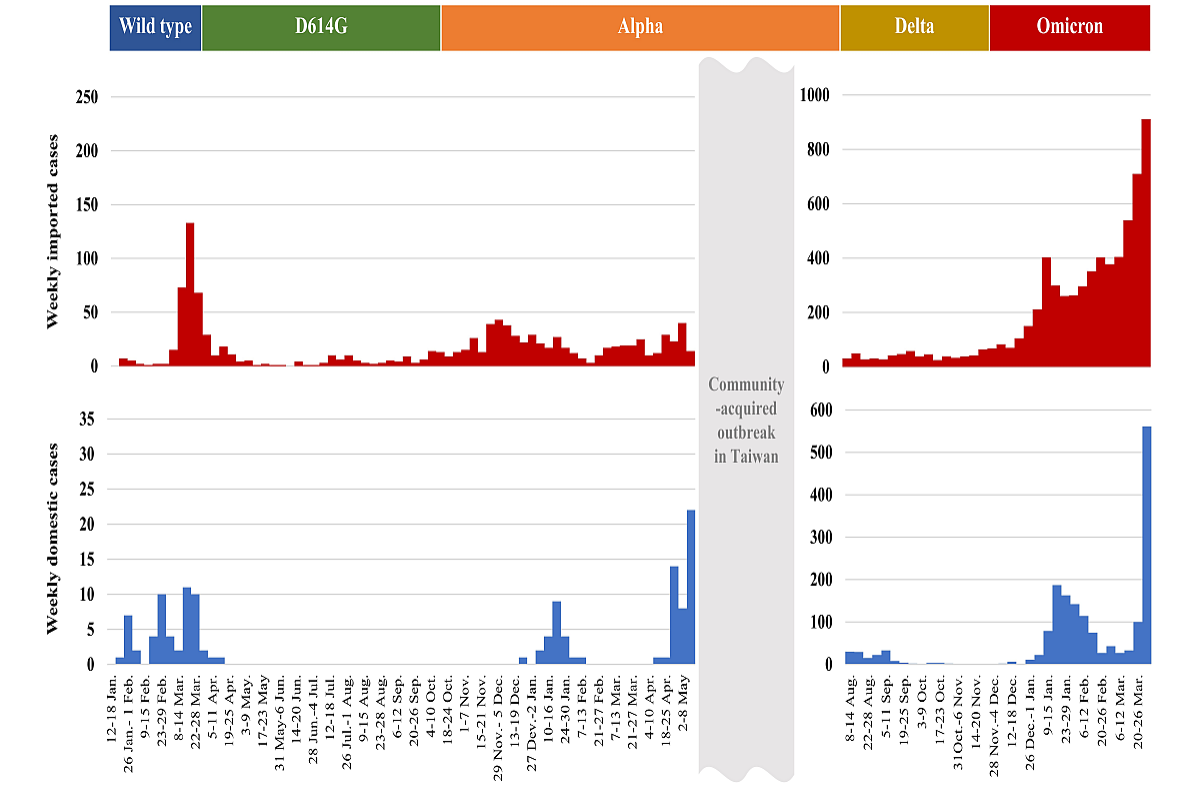
COVID-19 epidemics in Taiwan for periods without outbreaks by the origin of cases (imported vs domestic).

The containment measures for the non-VOC phase in Taiwan centered on 2 strategies, namely border control with quarantine and isolation, and NPIs without various strategies. Multimedia Appendix 1 shows the timelines of the evolution of border control measures for this non-VOC phase. The number of imported and domestic cases of COVID-19 by the date of onset on a weekly basis is presented in Figure 1.

The COVID-19 cases in Taiwan in 2020 mainly included imported cases (Figure 1). The risk of an outbreak following the transmission of COVID-19 to the community from these imported cases was largely reduced by the very strict border control strategies with quarantine and isolation in conjunction with NPIs, such as wearing masks and social distancing [19]. Multimedia Appendix 2 provides details on the criteria and guidelines for the implementation of 4 COVID-19 alert levels to target outbreaks in Taiwan.

### Containment Measures for the VOC Phase in Taiwan

The Alpha VOC became the predominant strain of the global pandemic by the end of 2020. Several cluster infections occurred in hospitals and households since January 2021, but were still under control until mid-May 2021, when a large-scale outbreak of the Alpha VOC occurred. On the top of border control measures with quarantine and isolation implemented since the non-VOC phase in Taiwan, the focus of containment measures for averting community-acquired outbreaks turned to community-based active surveillance with rapid test stations for the hotspots of outbreaks and enhanced NPIs, including strict regulation for wearing masks, restriction of public gathering, setting up of check points for high-risk areas such as public transportation sites and markets, and restriction of nonessential services such as restaurants and pubs. Multimedia Appendix 1 summarizes the timeline of the implementation of a series of containment measures for the VOC phase starting from the enhancement of NPIs from level 1 to level 2 alert until high restriction border control for travelers. The level 3 alert was rapidly extended to a nationwide level 3 alert on May 19, 2021 [20,21]. During the Delta VOC period (from August to December 2021) and Omicron VOC period (December 2021 onward), transmission in the community has been threatened by imported cases. In addition to containment measures, high coverage of vaccination has been an effective prevention strategy during these 2 periods. In response to the rapid spread of the Omicron VOC, inbound passengers have to follow updated regulations with more frequent reverse transcription-polymerase chain reaction (RT-PCR) testing plus rapid testing, and a possible mandatory 14-day quarantine based on the vaccination status. Additionally, inbound passengers have to provide negative COVID-19 RT-PCR test reports within 2 days and have to take a government-funded rapid RT-PCR test on arrival starting January 11, 2022. Owing to waning of the effects of vaccines, booster shots have been allowed for all adults who have received 2 vaccine doses for 12 weeks (84 days), since January 7, 2022.

### Statistical Analysis

#### New Surveillance Metrics for Quantifying Imported-Domestic Transmission

We used an extra-Poisson regression model with a Bayesian DAG approach [22] to calculate the expected weekly domestic cluster infections associated with imported cases of COVID-19, as shown in the right panel of Multimedia Appendix 3. For the *j*th county or city with the *Y*_tj_ domestic case at week *t*, the extra-Poisson regression model can be specified by

*Y*_jt_ ~ Poisson(μ_jt_),

log(μ_jt_)=offest_j_+α_j_+βX_j,t-1_

α_j_~Normal(α_0_, σ^2^_α_) **(1)**

where offest_j_ is the population of log scale, and the heterogeneity of imported-domestic transmission across counties and cities in Taiwan is captured by a normal distributed random intercept parameter, α_j_. While the common intercept parameter, α_0_, represents the common risk of transmission in Taiwan, the heterogeneity is captured by the variance parameter, σ^2^_α_. With this framework, the number of cases arising from domestic cluster infection caused by imported cases per week before (X_j,t-1_) can be assessed by using the regression coefficient β, which becomes the first surveillance metric and is denoted as Dci/Imc per week for estimating the effect size of domestic cluster infection. The larger the value of this metric estimated, the larger the domestic cluster infection. The extra variation across cities and counties regarding the transmission of COVID-19 associated with imported cases was captured by a random effect (α_j_) incorporated into the Poisson regression model, which is also called the random-effect Poisson regression model. The predicted distribution of the number of expected domestic cases in the next week (μ.pred[t+1]; Multimedia Appendix 3) can be generated by using the number of imported cases in the current week (X[t]; Multimedia Appendix 3) in conjunction with the posterior distribution of the force of transmission (β), standing for the metric of Dci/Imc per week, and the common intercept (α_0_) taking into account the county-level heterogeneity of COVID-19 transmission (σ^2^_α_). The Poisson model has been widely applied to sparse counts of domestic infection, which occur independently if there is a lack of larger cluster infections, with a high potential of developing into a large-scale community-acquired outbreak. If the observed value of our model is beyond the upper limit of the 95% credible interval (CrI), it means that sparse and independent assumptions based on the Poisson distribution are violated and implies a high potential of yielding a large-scale community-acquired outbreak. Accordingly, the second surveillance metric is to build up the alert threshold of emerging community-acquired outbreaks and to provide guidance for the rapid preparedness of containment measures (including effective and efficient contact tracing) for forestalling community-acquired outbreaks.

As mentioned above, data were divided into the non-VOC phase and VOC phase. The former period used for estimating the parameters of the following extra-Poisson regression model was based on imported and domestic cases between January 11 and June 20, 2020, covering the wild-type and D614G period in Taiwan. Because imported cases require an incubation time to generate secondary cases, we tested the lag time of imported cases by 0 weeks (concurrent, X_jt_), 1 week (X_j,t-1_), and 2 weeks (X_j,t-2_), and further selected the optimal lag time interval with the smallest deviance information criterion (DIC).

Regarding the impact of imported cases on the occurrence of domestic cases for the early Alpha (October 11, 2020, to May 12, 2021), Delta (August 8 to December 9, 2021), and Omicron (December 12, 2021, to April 2, 2022) VOC periods without outbreaks in Taiwan, a Bayesian negative binomial regression model was applied to take into account the heterogeneity across counties and cities associated with the imported-domestic transmission of COVID-19 owing to the lack of detailed information on the cases in counties and cities. Multimedia Appendix 4 shows the DAG model for assessing the force of imported-domestic transmission by using a Bayesian negative binomial regression model. Following the approach applied for the wild-type and D614G period, the 1-week lag model was adopted. For week *t*, the number of cases *Y*_t_ resulting from imported cases 1 week prior, *X*_t-1_, can be modeled by using the negative binomial regression model as follows:


Y_t_ ~ Negative Binomial (μ_t_, *k*),

log(μ_t_) = βX_t-1_
**(2)**

where the heterogeneity is captured by the dispersion parameter 1/*k*. Similar to the extra-Poisson regression model as above, the risk of imported-domestic transmission can thus be assessed by using the regression coefficient β for estimating the effect size of Dci/Imc. Following the extra-Poisson approach, the predicted distribution of the number of expected domestic cases in the next week (μ.pred[t+1]; Multimedia Appendix 4) for the Bayesian negative binomial model can be generated from the current number of imported cases (X[t]; Multimedia Appendix 4) by using the posterior distribution of imported-domestic transmission (β) and the dispersion parameter (1/*k*).

#### Estimation With the Bayesian MCMC Method

The Bayesian MCMC method was used to generate the samples derived from the posterior distributions of parameters for estimating 2 surveillance metrics. With the Markov chain underpinning, a stationary distribution for parameters can be reached in the long run under regular conditions. Independent samples can thus be generated from such a stationary posterior distribution on the basis of which inferences can be made [23]. The DAG models depicted in Multimedia Appendix 3 and Multimedia Appendix 4 were applied to facilitate the decomposition of joint distribution into full conditional density distribution by using the relationship between parent and child nodes [24]. Taking the extra-Poisson regression model as an example, the joint distribution,


P(Y, μ, α, α_0_, σ^2^_α_, β) **(3)**

is proportional to the product of the kernel distribution written by

*L*(Y, μ, α, α_0_, σ^2^_α_, β)=P(Y | μ)P(μ | **α**, β)P(**α** | α_0_, σ^2^_α_)P(β)P(α_0_)P(σ^2^_α_) **(4)**

In our application, noninformative priors were used to derive the samples from the stationary posterior distribution of parameters, including the risk of imported-domestic transmission (β), the common intercept (α_0_), and the county-specific random effect (σ_α_).

A block-wise Metropolic-Hasting sampler was applied to generate samples from the stationary posterior distribution. The sampling algorithm is detailed as follows:

1. Start with an initial value (β^(0)^, α^(0)^, α_0_^(0)^, σ^2^_α_^(0)^) selected from the support of each parameter.

2. Draw the candidate value for the first parameter, say β^(1)^, from a normal proposal distribution, *q*(β).

3. Compute the acceptance probability



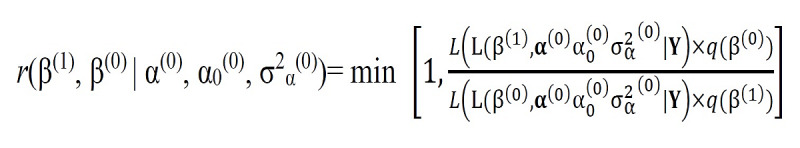



4. Draw *u* from uniform (0,1) and update β^(0)^ with β^(1)^ if *u* < *r*(β^(1)^, β^(0)^ | α^(0)^, α_0_^(0)^, σ^2^_α_^(0)^); otherwise, repeat steps 2 and 3.

5. Draw the candidate value for the next parameter, α^(1)^, to update the parameter sample with (α^(0)^ | α_0_^(0)^, σ^2^_α_^(0)^, β^(1)^) by using steps 2 to 4.

6. Repeat steps 2 to 5 for the rest of the parameters, (α_0_, σ^2^_α_), to derive (β^(1)^, α^(1)^, α_0_^(1)^, σ^2^_α_^(1)^) to complete an iteration of the update for parameter samples.

Thinning intervals of 10 and 100,000 iterations were used to generate the 10,000 posterior samples after 250,000 burn-in iterations by using the Bayesian MCMC methods mentioned above.

We estimated the effect size of Dci/Imc per week for each period corresponding to the type of SARS-CoV-2 variant. We built up the alert threshold by using the upper limit of the 95% CrI of the predicted number of domestic cases (μ.pred[t+1]; Multimedia Appendix 3 and Multimedia Appendix 4) generated by the parameters after updating the data on the non-VOC phase in Taiwan for alerting the possibility of yielding a large-scale community-acquired outbreak through imported-domestic transmission in the subsequent epochs. The possibility of a community-acquired outbreak was deemed low if observed domestic cases were not more than the alert threshold, namely the upper limit of the 95% CrI. Otherwise, an outbreak was likely to occur, and therefore, the rapid preparedness of containment measures, including effective and efficient contact tracing, would be flagged to forestall the ensuing community-acquired outbreak.

To validate the proposed surveillance model for the transmission from imported to domestic cases during the non-VOC period in Taiwan, the publicly available COVID-19 data provided by the Ministry of Health in New Zealand were used [25]. The chronological order of the incidence of COVID-19 for the hotspots was compared to validate the epidemic surveillance model for an outbreak.

All statistical analyses were performed using SAS 9.4 software (SAS Institute Inc).

## Results

### Evaluation of the Optimal Time Lag Model

After the application of the Bayesian MCMC method for the identification of the optimal time (in weeks) lag of imported cases prior to cases arising from domestic cluster infection, the 1-week lag of imported cases yielded a DIC of 255.8, which was smaller than the DICs of the model with concurrent week imported cases (260.3) and the model with a 2-week lag of imported cases (279.5).

### Surveillance Metrics for the Imported-Domestic Transmission Mode

**Figure 2:**
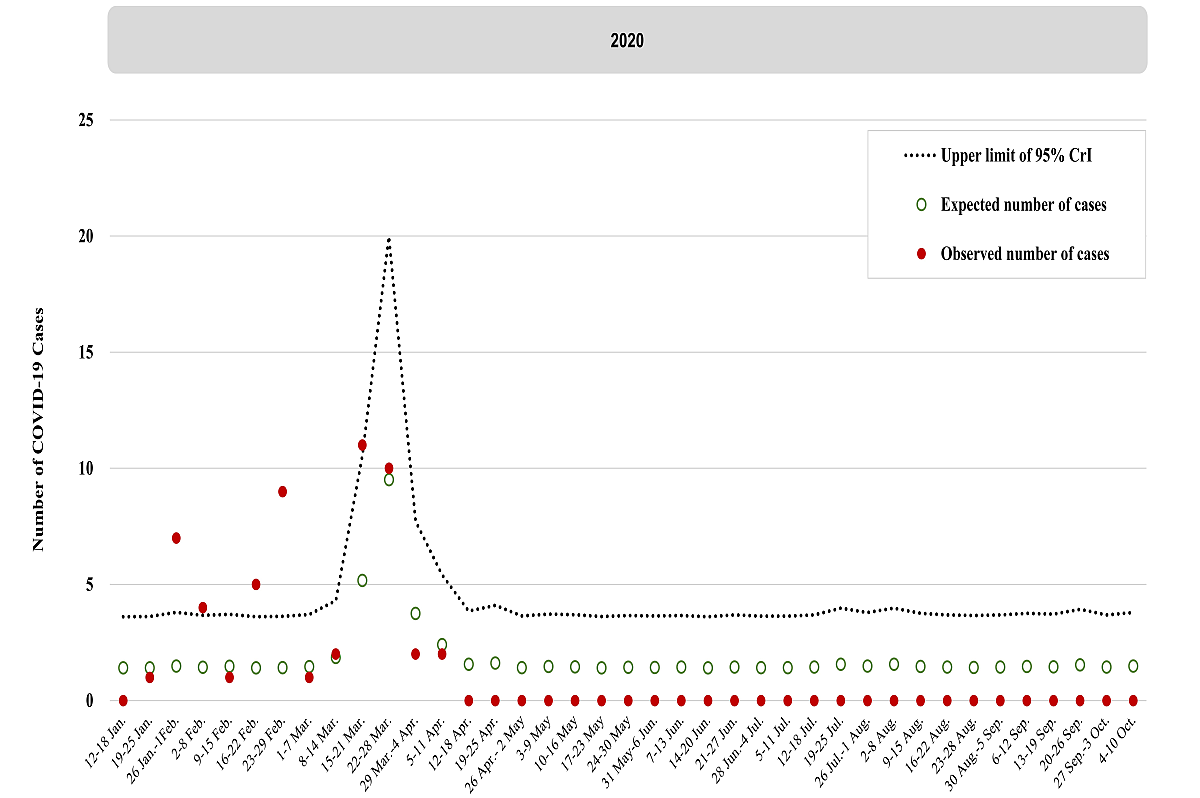
Number of observed (dotted point) and expected (green circle) domestic cases with the upper limit of the 95% credible interval (CrI) (dotted line) by week in the wild-type/D614G period.

The weekly observed number (red dot) and expected number (green circle) of domestic cases are shown in Figure 2 (wild-type and D614G period, January to September 2020), Figure 3 (Alpha VOC period, October 2020 to May 2021), and Figure 4 (Delta VOC period, mid-August to mid-December 2021; and Omicron VOC period, mid-December 2021 to early-April 2022). Table 1 shows the details of the estimated results of the parameters encoded in the Bayesian extra-Poisson regression model with a 1-week lag of imported cases regarding the 3 periods without outbreaks in Taiwan, namely the wild-type and D614G period, early Alpha VOC period, and Delta VOC period.

**Figure 3:**
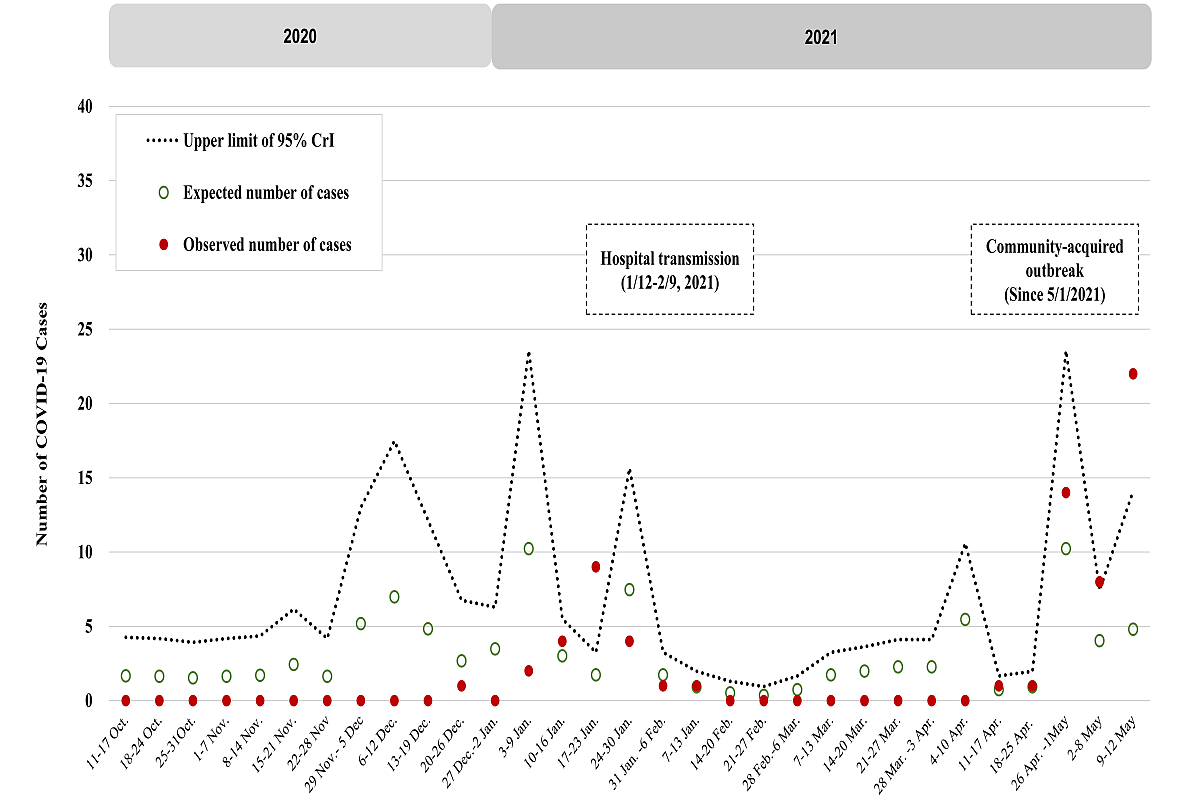
Number of observed (dotted point) and expected (green circle) domestic cases with the upper limit of the 95% credible interval (CrI) (dotted line) by week in the Alpha variant of concern period.

**Figure 4:**
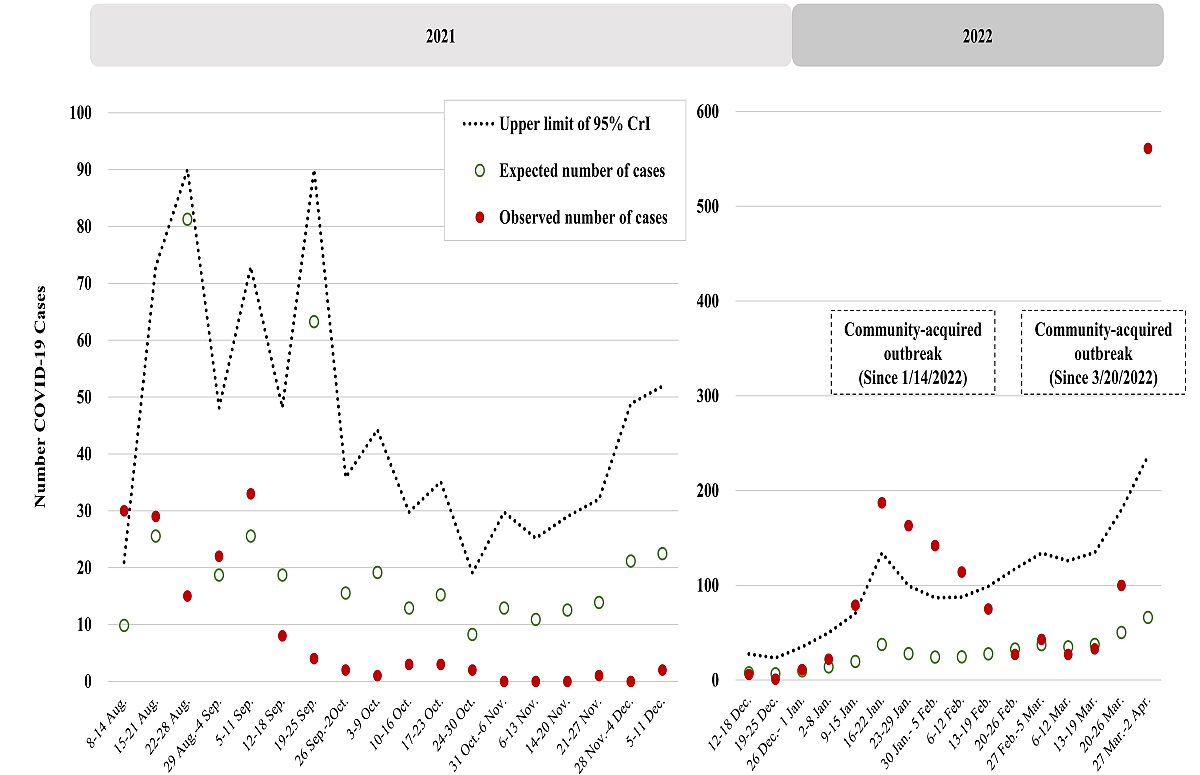
Number of observed (dotted point) and expected (green circle) domestic cases with the upper limit of the 95% credible interval (CrI) (dotted line) by week in the Delta (August 8, 2021, to December 9, 2021) and Omicron (December 12, 2021, to April 2, 2022) variant of concern periods.

The upper bound of the 95% CrI of expected cases (dotted line, Figures 2-4) has been plotted to provide the alert threshold of domestic cluster infection in the community caused by transmission from imported cases 1 week before. This 1-week prior alert on the risk of elevated Dci/Imc per week guided the vigilance on NPIs for averting further community-acquired outbreaks.

**Table 1:** Estimated results for the risk of imported-domestic transmission of COVID-19 for 3 periods in Taiwan.

Parameter			Estimate	95% CI
**Wild-type/D614G period (January to September 2020)**				
	Common intercept		−3.5457	−5.1978 to −2.4413
	Risk of imported-domestic transmission		0.0954	0.0644 to 0.1259
	Standard error of random intercept, *σ_ν_*		1.8116	0.9861 to 3.7359
**Alpha VOC^a^ period (October 2020 to May 2021)**				
	Intercept		−1.9448	−4.1238 to 0.0712
	Risk of imported-domestic transmission		0.1414	0.0541 to 0.2510
	Dispersion parameter		1.7438	0.2734 to 3.8218
**Delta VOC period (August to December 2021)**				
	Risk of imported-domestic transmission		0.1005	0.0685 to 0.1332
	Dispersion parameter		1.5454	0.3954 to 3.3562
**Omicron VOC period**				
	**December 2021 to January 2022**			
		Risk of imported-domestic transmission	0.0459	0.0366 to 0.0575
		Dispersion parameter	1.0054	0.4192 to 2.2245
	**February to April 2022**			
		Risk of imported-domestic transmission	0.0429	0.0352 to 0.0519
		Dispersion parameter	1.5969	0.8484 to 2.8928

^a^VOC: variant of concern.

#### Wild-Type and D614G Period

During the wild-type/D614G period, the estimated Dci/Imc per week was 9.54% (95% CrI 6.44%-12.59%; Table 1). Figure 2 shows that there were 5 weeks (January 26 to February 1, February 2 to February 8, February 16 to February 22, February 23 to February 29, and March 15 to March 21, 2020) in which the observed numbers of domestic cases exceeded the alert thresholds. This period yielded 81% of clustered cases (22 of 27 community-acquired cases) in 5 clusters in Taiwan, including 3 household clusters (with 5, 3, and 6 COVID-19 cases, respectively), 1 medical institute cluster (with 9 COVID-19 cases), and 1 academic institute cluster (with 4 COVID-19 cases). Guidance of the alert thresholds from this early period of the wild-type COVID-19 strain provided a strong rationale for being on alert for the ensuing cluster infections from the preceding 1 week when imported cases were introduced. This accounted for why none of these 5 cluster events led to any large-scale community-acquired outbreaks in Taiwan. There was rapid preparedness of containment measures with strict NPIs together with effective and efficient contact tracing of all possible susceptible individuals.

Figure 2 shows that this surveillance metric was very useful, particularly when there was a substantial surge in imported cases resulting from the large-scale COVID-19 pandemic worldwide. This could be seen in our cases between March and April 2020, as shown in Figure 1. Again, the surveillance metric was used for alerting about possible cluster infections to forestall further community-acquired outbreaks. Alerted by the threshold (20 domestic cases per week), the observed domestic cases were kept lower than the alert threshold to avoid large-scale outbreaks in April. Since then, there had not been any domestic case until December 2020.

#### Alpha VOC Period

**Figure 5:**
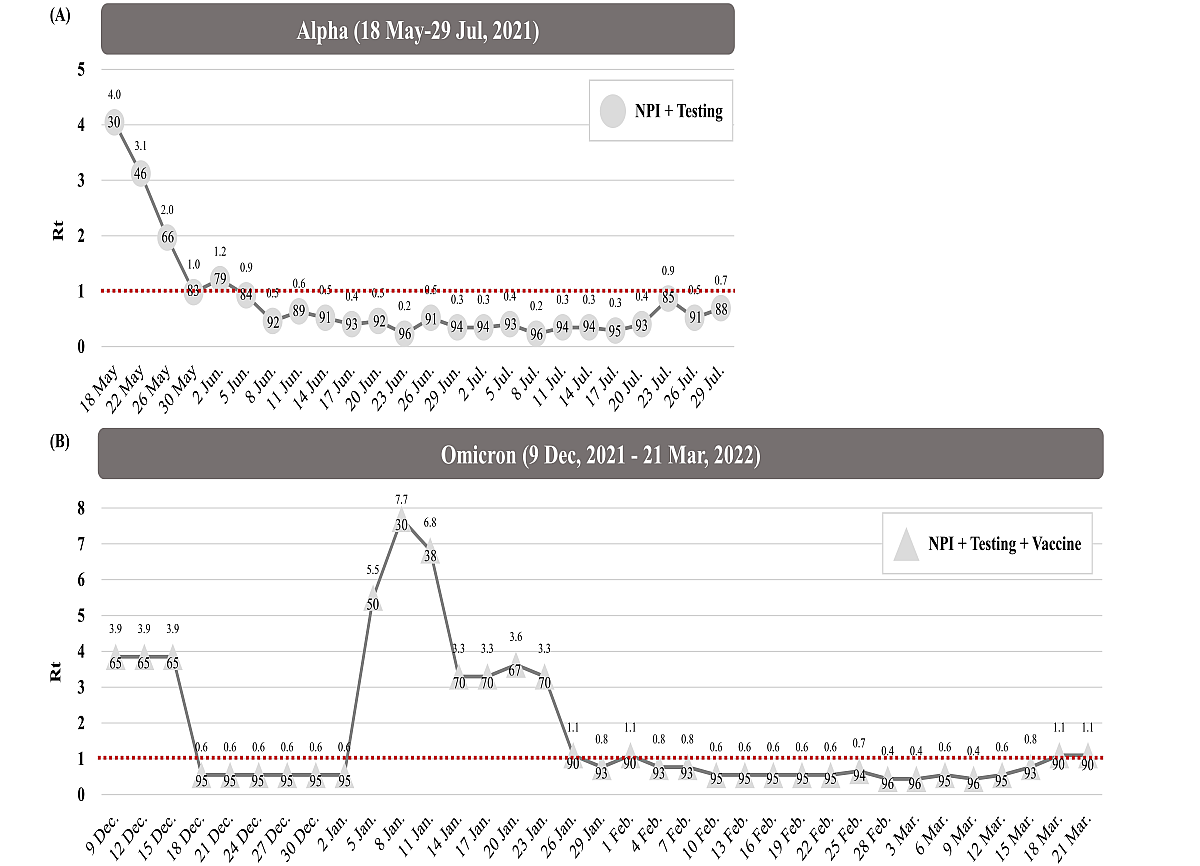
Effective reproductive number (Rt) and effectiveness of nonpharmaceutical interventions (NPIs) and testing in Taiwan. (A) Alpha variant. (B) Omicron variant.

There had been no outbreak during the early Alpha VOC phase of the COVID-19 pandemic from October to December 2020. The second surge of imported cases occurred from January 2021 onwards (Figure 1). Again, there was 1 week (January 17 to January 23, 2021) in which the observed number of domestic cases was beyond the alert threshold, resulting from hospital-based cluster infections and 3 subsequent household clusters (11 family members) (Figure 3). The source of this cluster infection was later identified as an imported case infected with the Alpha VOC. After being alerted by the proposed surveillance metric and following timely contact tracing and containment measures, including quarantine and isolation for all staff members in the hospital and their close contacts for 14 days, there was no further outbreak until early May 2021, when the number of observed domestic cases reached beyond 20, which was higher than the expected surveillance curve (5 cases) and the corresponding upper bound of the 95% CrI (14 cases). The estimated results on the basis of the empirical data during this Alpha VOC period showed that the Dci/Imc increased to 14.14% (95% CrI 5.41%-25.10%; Table 1). The peak of domestic cases far beyond the threshold value in early May 2021 not only presaged the ensuing outbreaks, but also revealed the loose NPIs at that time without an available vaccine in Taiwan. If effective contact tracing and timely containment measures had been deployed in advance on the basis of the increased risk and the alert threshold in the week between May 2 and May 8, the ensuing community-acquired outbreak involving the Alpha VOC could have been prevented (bottom panel of Figure 1). This outbreak lasted for 2.5 months and subsided in July 2021. The corresponding periods of the estimated results for the effectiveness of NPIs and testing implemented in Taiwan during the Alpha VOC phase are shown in Figure 5A.

#### Delta and Omicron VOC Period

After controlling the outbreak in the Alpha VOC period, there was monitoring of domestic cluster infections for the Delta VOC since August 8, 2021, and the Omicron VOC from December 10, 2021. After mid-August, there had been a cluster infection during the Delta VOC phase of the COVID-19 pandemic. The estimated results (Table 1, Delta VOC period) showed that the Dci/Imc remained at 10.01% (95% CrI 6.85%-13.32%), although the Delta VOC had higher transmissibility and escaped vaccine-induced immunity. The left panel of Figure 4 shows that for 2 weeks (August 22 to August 28, 2021, and September 19 to September 25, 2021) when a surge in the number of domestic cases as a result of Dci/Imc was expected (green circle), the observed number of domestic cases was far below the threshold of outbreak. This can be attributed to the implementation of enhanced containment measures, including the strengthening of border control strategies with multiple tests on arrival and during quarantine, the collective quarantine strategy, and the elevated alerts of NPIs to levels 2 and 3 since the outbreak in May 2021 in Taiwan. Since then, the weekly observed cases were below the alert threshold owing to NPIs with a level 2 alert and the rapid administration of vaccines.

The right panel of Figure 4 shows the observed and predicted numbers of cases along with the alert threshold during the Omicron VOC period. Given the increasing coverage rate of vaccination, the risk of domestic cluster infection per imported case for the Omicron BA.1 VOC reduced to 4.59% (95% CrI 3.66%-5.75%), but an upsurge in domestic cases was still observed because the Omicron BA.1 VOC was considered to have a high transmission probability. In the week from January 9 to January 15, 2022, the observed number exceeded the alert threshold, indicating a high potential for a community-acquired outbreak. There was indeed a small-scale community-acquired outbreak of the Omicron BA.1 VOC. After a series of containment measures, including rapid RT-PCR tests for inbound passengers on arrival coupled with stringent quarantine and isolation, rapid booster vaccination, and enhanced NPIs with a level 2 alert in the community, the community-acquired outbreak subsided by the end of February 2022 (Figure 5).

There was a return to the imported-domestic transmission model, with the surveillance metric of Dci/Imc estimated at 4.29% (95% CrI 3.52%-5.19%) from February until March 20, 2022. After that, a similar circumstance beyond the alert threshold was noted for the invasion of the imported Omicron BA.2 VOC, and a community-acquired outbreak started from March 20 to 26, 2022, resulting in a large-scale community-acquired outbreak from early April until July 2022.

### External Validation of the Surveillance Metrics for Domestic Cluster Infections Using Imported Cases in New Zealand

To validate the proposed model and extend its application to different periods of SARS-CoV-2 variants, the proposed extra-Poisson regression model was applied to data on the New Zealand COVID-19 outbreak in 2020. Multimedia Appendix 5 shows the estimated results obtained. Notably, in New Zealand, the risk of Dci/Imc per week increased to 9.38% (95% CrI 8.88%-9.86%), which was close to the estimated results based on Taiwan data (9.54%, 95% CrI 6.44%-12.59%) in the same period. Details regarding the spatial temporal distribution of COVID-19 outbreaks by types of cases in New Zealand are provided in Multimedia Appendix 6. Multimedia Appendix 7 shows the predicted number of domestic cases by using the parameters trained from the empirical data of New Zealand (Multimedia Appendix 5). Similar to the application in Taiwan, the risk of an outbreak associated with imported cases could be assessed by comparing the observed cases (red dot in Multimedia Appendix 7) with the alert threshold (dotted line in Multimedia Appendix 7). The detailed interpretation of the results of this external validation is elaborated in Multimedia Appendix 8.

## Discussion

Many cyclical community-acquired outbreaks in each country or region during the COVID-19 pandemic have been noted from 2020 to 2022, and these epidemics have occurred in parallel with the evolution of various emerging SARS-CoV-2 variants, including the wild-type/D614G strain during the non-VOC phase and the Alpha, Beta, Gamma, Delta, and Omicron strains during the VOC phase. More importantly, when facing emerging variants, there were corresponding chains of containment measures with the following 3 serial steps: (1) border control of imported cases together with quarantine and isolation; (2) contact tracing and epidemic investigation of domestic cluster infection together with testing for detecting the foci of infection earlier (small households to large institutions); and (3) control of large-scale community-acquired infection with population-based approaches, mainly involving NPIs and mass vaccination. Although most countries focus on steps 1 and 2 for averting community-acquired outbreaks in the beginning, they end up having no choice but to adopt step 3 involving population-based approaches. Accordingly, most epidemic surveillance models still follow traditional surveillance metrics like the effective basic reproductive number (R_t_) for containing community-acquired outbreaks. However, it is still important to develop a new surveillance model with new surveillance metrics commensurate with steps 1 and 2 for forestalling community-acquired outbreaks when facing emerging SARS-CoV-2 variants like the updated Omicron subvariants BA.4/BA.5. Most importantly, such a new surveillance model with useful metrics can be robustly applied across countries and time, covering various emerging SARS-CoV-2 variants detected from imported cases.

Using Taiwan empirical data, the proposed new surveillance model for monitoring cluster infections in the wake of imported cases was assessed in 5 periods (wild-type, D614G, Alpha VOC, Delta VOC, and Omicron VOC). The first metric of Dci/Imc per week was used to estimate the effect size of the risk of infection through imported-domestic transmission. The second metric involving the upper bound of the 95% CrI for predicted domestic cases of cluster infections derived from imported cases 1 week before provided the alert threshold for guiding the preparedness of containment measures for preventing community-acquired outbreaks in each country or region. Such an alert threshold would be affected by the characteristics of each emerging SARS-CoV-2 variant, as well as the underlying coverage rate of vaccination and the extent of NPIs. By using empirical data on imported and domestic COVID-19 cases in Taiwan, the effect size of Dci/Imc and the alert threshold were estimated as 9.54% and 12.59% for the wild-type/D614G strain, 14.14% and 25.10% for the Alpha VOC, 10.05% and 13.32% for the Delta VOC, 4.59% and 5.75% for the Omicron BA.1 VOC, and 4.29% and 5.19% for the Omicron BA.2 VOC, respectively, in 2 periods. It should be noted that the interpretation of the absolute effect size of Dci/Imc across various emerging SARS-CoV-2 variants should be taken with great caution.

When a similar logic is applied to the alert threshold, the threshold value for weekly domestic cases during 3 SARS-CoV-2 variant periods would not go beyond 20 cases under the low coverage rate of vaccination and good performance of NPIs before the Omicron VOC period. After the Omicron VOC period, the alert threshold for weekly domestic cases would be 180 cases under the high vaccination rate and minimal NPIs. Empirical evidence on whether and how community-acquired outbreaks can be averted through different periods of SARS-CoV-2 variants with the proposed new surveillance model has been demonstrated by Taiwan data. Outbreaks were averted during the wild-type and D614G periods. In contrast, large-scale outbreaks could not be averted during the Alpha VOC period when the expected number of domestic cases was far beyond the alert threshold for an outbreak between May 9 and May 12, 2021, because of the increased transmissibility of the Alpha VOC that was supported by the increased risk of imported-domestic transmission in comparison with the wild-type/D614G variant (14.14% vs 9.54%). However, a low level of NPIs might also have contributed to such an outbreak around mid-May 2021 (30%; Figure 5). In the Delta VOC period after excluding the outbreak related to the Alpha VOC in Taiwan, the level of the NPI alert and the strict border control strategies implemented since the outbreak period reduced the risk of imported-domestic transmission to 10.05% (Table 1) and averted a community-acquired outbreak of the Delta VOC.

Given the high coverage of full vaccination, lower estimates of Dci/Imc for the Omicron VOC were seen, ranging from 4.3% to 4.6%. As the protective effect of the Oxford/AstraZeneca vaccine in particular started to wane in the community, the observed number of domestic cases was beyond the threshold of outbreak during the early Omicron BA.1 VOC period. Guided by the alert threshold, several containment strategies, including more restricted border control and rapid RT-PCR testing on arrival for travelers, and rapid booster shots for eligible adults, were implemented to avert a large-scale outbreak. However, another large-scale community-acquired outbreak could not be averted in late March 2022 owing to the observed cases going beyond the alert threshold partially due to waning of the protective effect of the mRNA-1273 vaccine (Moderna) or BNT162b2 vaccine (Pfizer–BioNTech).

Although our metrics of the risk for domestic cluster infection and the alert threshold are pivotal in imported cases 1 week before applying the surveillance model for monitoring imported cases 1 week prior to the formation of cluster infections of domestic COVID-19 cases, they are very useful for alerting the surrounding community in proximity to imported cases beyond the threshold of the upper bound of the 95% CrI to enhance NPIs and active rapid testing with effective contact tracing and epidemic investigation for the observed cases. This accounted for the lack of community-acquired outbreaks before mid-May 2021 in Taiwan. Such good control over the COVID-19 epidemic has been reported in previous studies by evaluating NPIs at the individual and population levels [5,19,26], and using the traditional surveillance model for assessing the duration from R_t_ larger than 1 to R_t_ smaller than 1 and the case load following the machining learning model [27].

Several previous studies have proposed an early warning model in relation to contact tracing and epidemic investigation before community-acquired outbreaks. However, our study differs from 2 recent previous studies [15,16] that developed an early warning model of COVID-19 outbreaks, in 2 main aspects. First, both studies covered a short period that reflected 1 or 2 SARS-CoV-2 strains and used data based on a single country. They were therefore unable to test the robustness of their models for a series of SARS-CoV-2 variants and samples across countries. Guan et al used human mobility data in Israel over the period from February 1, 2020, to January 7, 2021. Specifically, they trained their model over the period from April 6 to October 24, 2020, and evaluated the model’s predictive ability over 2 very short periods (November 1-30, 2020, and December 1-31, 2021) [15]. Kogan et al employed data in the United States that had been obtained from multiple digital traces over 2 short periods (March 1-May 31, 2020, and June 1-September 30, 2020) [16]. In contrast, the proposed new surveillance model made use of the full chronological empirical data in Taiwan from January 1, 2020, to April 2, 2022, covering various emerging SARS-CoV-2 variants. Our model is robust across a long period involving various variants and across countries covering different geographical and cultural conditions when using imported cases. Second, the data derived from digital traces, for example, Google Trends, used in the studies by Guan et al and Kogan et al may be affected by media activities. Furthermore, it is not easy to obtain accurate real-time data in countries with unavailable technological infrastructure, strong information censoring, and a lack of transparency. Instead of focusing on digital traces, we made use of imported cases that may be less likely to be affected by confounding factors. The use of imported data in our proposed surveillance model can be applied to countries with different political and social conditions and at different technological development stages. Moreover, our study is highly relevant to health regulators and public health policy makers, particularly in countries that have opened their borders and eased/removed NPI measures.

In addition to the illustration of the Taiwan experience, the external validation involving New Zealand further adds credibility to the application of this surveillance model in a scenario without an outbreak. This model can also be applied to those countries with controllable community-acquired outbreaks, such as Israel [28] and Qatar [29], after the mass vaccination program since early 2021, to monitor the impact of imported cases on the risk of domestic cluster infection. This is especially important for outbreaks resulting from vaccine breakthrough in countries or regions with high vaccine coverage, such as Singapore [30] and Israel [31], or the rapid waning of booster effectiveness worldwide, possibly affecting the community-level spread of SARS-CoV-2 VOCs [32].

One of the major limitations of the current model pertains to the generalization of the proposed new surveillance model. There are 2 major circumstances that require the refinement of the current proposed epidemic surveillance model. The proposed model has not incorporated health care capacity for accommodating the threshold of tolerable COVID-19 cases responsible for each episode of the outbreak. In consideration of resuming prepandemic activity, making allowance for this factor is of paramount importance for the implementation of NPIs and testing given the vaccine coverage rate. Different countries and regions may require different outbreak thresholds based on this global surveillance model. With increasing cases of vaccine breakthrough; the rapid emergence of VOCs with a wide spectrum of immunogenicity, high transmissibility, and resistance to antibodies associated with natural infection or vaccination; and the waning of immunity in the population, there is a high likelihood for the continued spread of SARS-CoV-2 in the population [33]. Given the possibility of a long-term association between SARS-CoV-2 and the human population, the goal of epidemic surveillance may shift from the elimination of this pathogen to a balance among health care capacity, socioeconomic activity, and population immunity. If this occurs, the proposed surveillance model should take this factor into account and should be used as a guide to inform the containment measures required to mitigate large-scale outbreaks according to health care capacity. Moreover, as the border control policy on quarantine and isolation of imported cases gets altered with the advent of high-performance rapid testing and the gradual expansion of vaccine coverage worldwide, the surveillance model for monitoring imported-domestic transmission to avert outbreaks may vary from country to country, depending on the extent of NPIs, administration of tests, coverage rate of vaccines, and administration of vaccine boosters. Such a heterogeneity should be taken into account to refine the surveillance model on imported-domestic transmission when it is applied to avert a large-scale outbreak. More importantly, our new surveillance model and metrics are not meant to replace conventional surveillance and corresponding metrics like R_t_ for assessing how to eliminate the spread of large-scale community-acquired outbreaks. When a community-acquired outbreak occurs, the conventional surveillance SEIR model is needed to assess the effectiveness of containment measures, as shown in Figure 5. Based on the SEIR model, the R_t_ decreased from 4.0 to 0.7 from May 18, 2021, to July 31, 2021. The effectiveness of NPIs and testing, which reflects the strategies implemented 2 weeks ago, was over 60% after May 26, 2021, and increased to over 90% after June 14, 2021. A similar finding was noted for a community-acquired outbreak of Omicron BA.2. Again, R_t_ reduced from 7.7 (value of R_t_ in early January) to less than 1 (around the end of January; Figure 5B). Our proposed new surveillance model has a supplementary role as a global vigilance method for forestalling large-scale local community-acquired outbreaks of emerging SARS-CoV-2 VOCs in each country and region worldwide.

In conclusion, a global new surveillance model and metrics have been proposed for monitoring imported cases of SARS-CoV-2 variants from the non-VOC phase to the VOC phase, using the Taiwan scenario. The new surveillance model and metrics are very useful for forestalling a new large-scale community-acquired outbreak through monitoring of the imported-domestic transmission mode associated with emerging infectious diseases in the future.

## Appendix

Multimedia Appendix 1 Timelines of the SARS-CoV-2 variant of concern outbreak and the implementation of key containment measures in Taiwan.

Multimedia Appendix 2 Criteria and guidelines for the 4 COVID-19 alert levels in Taiwan.

Multimedia Appendix 3 Directed acyclic graphic model of the Bayesian extra-Poisson regression model for assessing the force of imported-domestic transmission.

Multimedia Appendix 4 Directed acyclic graphic model of the Bayesian negative binomial model for assessing the force of imported-domestic transmission.

Multimedia Appendix 5 Estimated results for the risk of the imported-domestic transmission of COVID-19 in New Zealand with the consideration of heterogeneity across counties using the Poisson model.

Multimedia Appendix 6 Epidemic curve of the COVID-19 outbreak in New Zealand by types of cases.

Multimedia Appendix 7 Number of observed (blue dot) and expected (green circle) domestic cases with the upper limit of the 95% credible interval (dotted line) by week.

Multimedia Appendix 8 Surveillance metrics for domestic cluster infections using imported cases in New Zealand.

## Abbreviations

CrI: credible interval
DAG: directed acyclic graphic
Dci/Imc: number of cases arising from domestic cluster infection caused by imported cases
DIC: deviance information criterion
MCMC: Markov Chain Monte Carlo
NPI: nonpharmaceutical intervention
Rt: effective reproductive number
RT-PCR: reverse transcription-polymerase chain reaction
SEIR: Susceptible-Exposed-Infected-Recovery
VOC: variant of concern
