# Supplementary material for: New Surveillance Metrics for Alerting Community-Acquired Outbreaks of Emerging SARS-CoV-2 Variants Using Imported Case Data: Bayesian Markov Chain Monte Carlo Approach
Source: JMIR Public Health Surveill. 2022 Nov 25;8(11):e40866. doi: 10.2196/40866 (PMC9746786; doi:10.2196/40866)

**Multimedia Appendix 1.** Timelines of the SARS-CoV-2 variant of concern outbreak and the implementation of key containment measures in Taiwan.


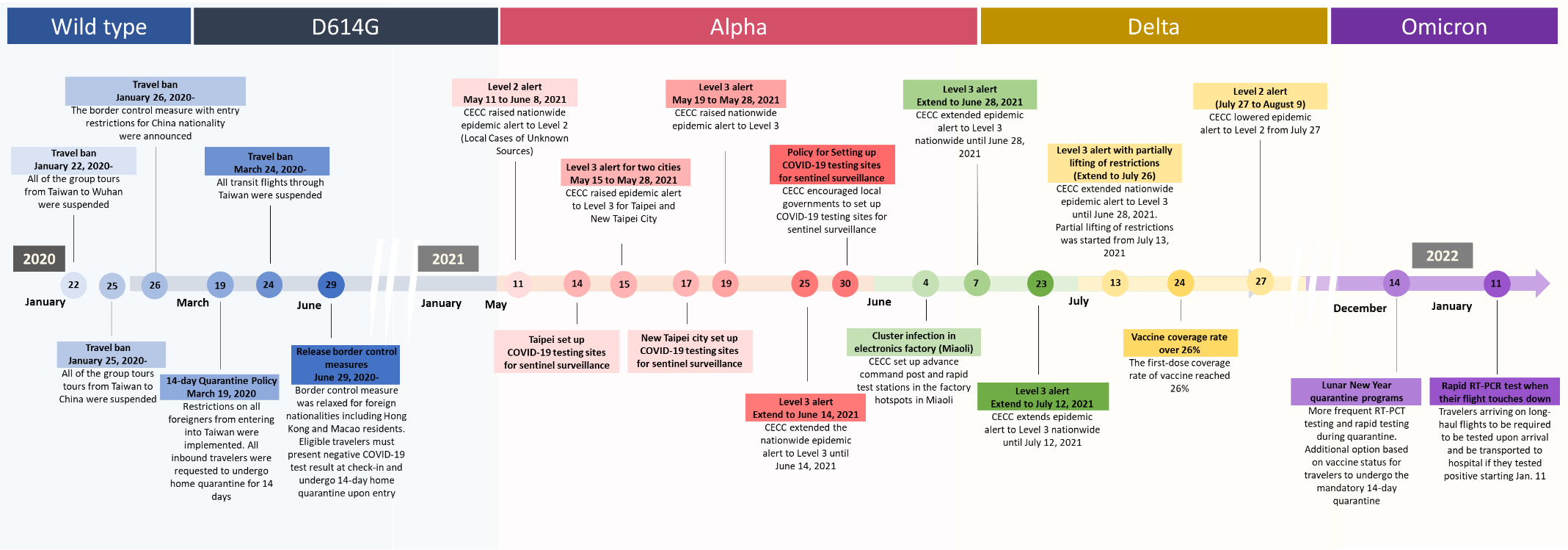

Supplement: Multimedia Appendix 1 [file publichealth_v8i11e40866_app1.docx]
