# Supplementary material for: New Surveillance Metrics for Alerting Community-Acquired Outbreaks of Emerging SARS-CoV-2 Variants Using Imported Case Data: Bayesian Markov Chain Monte Carlo Approach
Source: JMIR Public Health Surveill. 2022 Nov 25;8(11):e40866. doi: 10.2196/40866 (PMC9746786; doi:10.2196/40866)
Supplement: Multimedia Appendix 2 [file publichealth_v8i11e40866_app2.docx]

**Multimedia Appendix 2.** Criteria and guidelines for the 4 COVID-19 alert levels in Taiwan.

| **Level** | **Criteria** | **Guidelines** |
| --- | --- | --- |
| **Level 1** | Imported cases resulting in isolated community transmission | • Wearing mask on public transportation and in crowded public venues is encouraged.  • The CECC recommends the cancellation or postponement of non-essential gatherings that will bring people into close contact with others.  • Businesses and public venues required to implement real-name registration, social distancing, temperature checks, and regular disinfection |
| **Level 2** | Domestically transmitted cases from unknown sources. | • Fines imposed for those failure to follow mask guidelines.  • Outdoor gatherings over 500 and indoor gatherings over 100 banned.  • Public gatherings must implement social distancing, mask-wearing/partitions, an identification-based registration system, temperature checks, crowd controls and routine disinfection or be cancelled.  • Places of business must impose crowd controls; those unable to implement necessary epidemic prevention measures should temporarily suspend operations.  • When necessary, the CECC may order the closure of entertainment or leisure-related businesses or public venues. |
| **Level 3** | Three community clusters within a week (or) ten domestically transmitted cases from unknown sources in one day. | • Wearing mask at all times outdoors is mandatory.  • Cancellation of outdoor gatherings of 10+ people and indoor gatherings of 5+ people.  • All places of business and public venues are shuttered, with the exception of essential services, police departments, hospitals, and government buildings.  • At places of business or public venues that remain open, mask wearing and social distancing is mandatory.  • In areas where community transmission has taken place, residents are restricted to a set perimeter and must submit to COVID-19 testing. |
| **Level 4** | Sharp increase in domestic cases (a daily average of above 100 cases over the last 14 days) with at least half transmitted from unknown sources. | • People can only leave home for essential activities (ex. to purchase food, receive medical treatment, or for essential work) and must wear a mask and maintain a social distance at all times.  • Masking and social distancing must be practiced at home.  • All public events cancelled.  • Apart from essential services, law enforcement, medical and government services, all in-person work and school are suspended.  • Lockdowns will be imposed in counties, cities, or townships where an outbreak has become severe. Only designated persons may enter or leave these lockdown zones, and residents must stay in their homes |
