# Supplementary material for: New Surveillance Metrics for Alerting Community-Acquired Outbreaks of Emerging SARS-CoV-2 Variants Using Imported Case Data: Bayesian Markov Chain Monte Carlo Approach
Source: JMIR Public Health Surveill. 2022 Nov 25;8(11):e40866. doi: 10.2196/40866 (PMC9746786; doi:10.2196/40866)

**Multimedia Appendix 4.** Directed acyclic graphic model of the Bayesian negative binomial model for assessing the force of imported-domestic transmission.


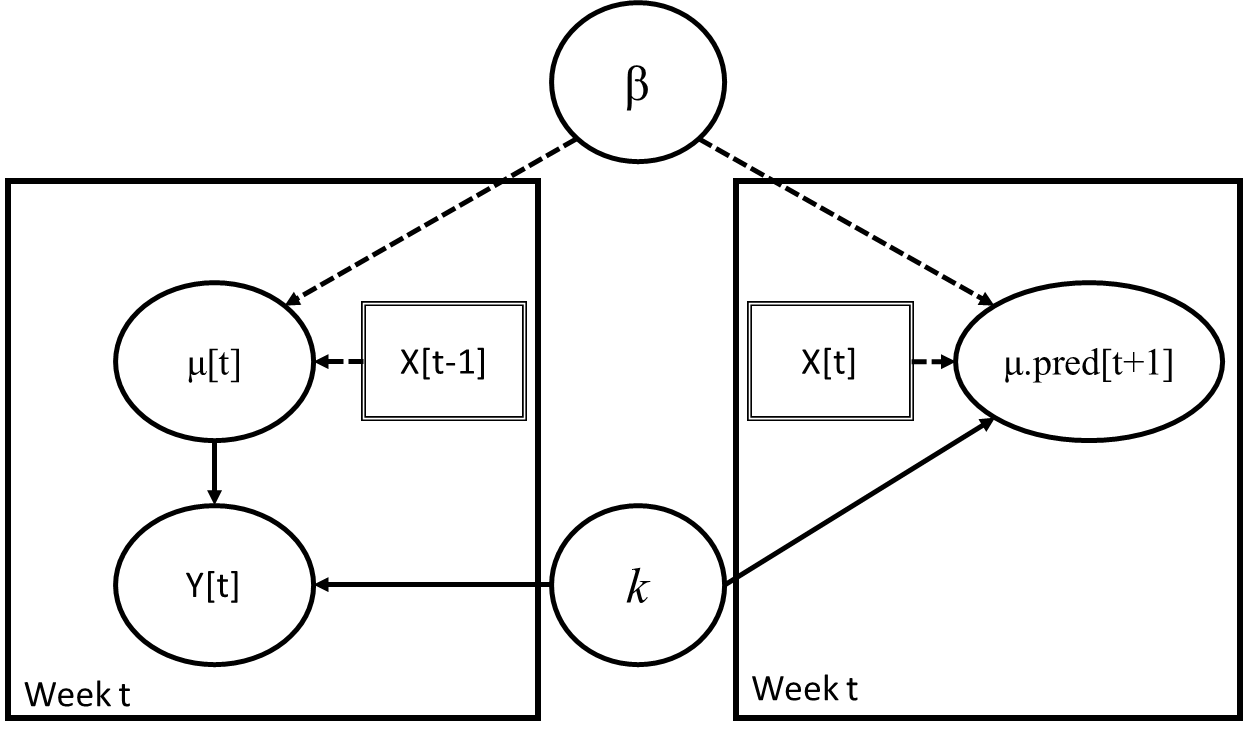

Supplement: Multimedia Appendix 4 [file publichealth_v8i11e40866_app4.docx]
