# Supplementary material for: New Surveillance Metrics for Alerting Community-Acquired Outbreaks of Emerging SARS-CoV-2 Variants Using Imported Case Data: Bayesian Markov Chain Monte Carlo Approach
Source: JMIR Public Health Surveill. 2022 Nov 25;8(11):e40866. doi: 10.2196/40866 (PMC9746786; doi:10.2196/40866)
Supplement: Multimedia Appendix 5 [file publichealth_v8i11e40866_app5.docx]

**Multimedia Appendix 5.** Estimated results for the risk of the imported-domestic transmission of COVID-19 in New Zealand with the consideration of heterogeneity across counties using the Poisson model.

| Parameter | Estimate | 95% CI |
| --- | --- | --- |
| Common intercept | -0.2409 | -0.8362 to 0.3069 |
| Risk of imported-domestic transmission | 0.0938 | 0.0888 to 0.0986 |
| Standard error of random intercept, σ_ν_ | 1.1987 | 0.8393 to 1.8004 |
