# Supplementary material for: New Surveillance Metrics for Alerting Community-Acquired Outbreaks of Emerging SARS-CoV-2 Variants Using Imported Case Data: Bayesian Markov Chain Monte Carlo Approach
Source: JMIR Public Health Surveill. 2022 Nov 25;8(11):e40866. doi: 10.2196/40866 (PMC9746786; doi:10.2196/40866)
Supplement: Multimedia Appendix 6 [file publichealth_v8i11e40866_app6.docx]

**Multimedia Appendix 6.** Epidemic curve of the COVID-19 outbreak in New Zealand by types of cases.

3/14 Compulsory household isolation and quarantine extending to other areas

3/20 Traveling ban for foreigners

2/1 Border control and compulsory household isolation and quarantine for China

3/25 Lock down for four weeks
