# Supplementary material for: New Surveillance Metrics for Alerting Community-Acquired Outbreaks of Emerging SARS-CoV-2 Variants Using Imported Case Data: Bayesian Markov Chain Monte Carlo Approach
Source: JMIR Public Health Surveill. 2022 Nov 25;8(11):e40866. doi: 10.2196/40866 (PMC9746786; doi:10.2196/40866)
Supplement: Multimedia Appendix 7 [file publichealth_v8i11e40866_app7.docx]

**Multimedia Appendix 7.** Number of observed (blue dot) and expected (green circle) domestic cases with the upper limit of the 95% credible interval (dotted line) by week.

**Auckland August Cluster (8/11-8/29)**

138 domestic cases

**Number of Cases >20 (3/22-3/28)**

Southern (36),

Waikato (25), Auckland (27)

**Number of Cases >20 (4/5-4/11)**

Southern (42), Waitematā (38), Auckland (34), Canterbury (34), Waikato (26)

**Number of Cases >20 (3/29-4/4)**

Southern (72), Waikato (68), Waitematā (50), Canterbury (42), Counties Manukau (29),

Auckland (25), Capital and Coast (21)
