# Supplementary material for: New Surveillance Metrics for Alerting Community-Acquired Outbreaks of Emerging SARS-CoV-2 Variants Using Imported Case Data: Bayesian Markov Chain Monte Carlo Approach
Source: JMIR Public Health Surveill. 2022 Nov 25;8(11):e40866. doi: 10.2196/40866 (PMC9746786; doi:10.2196/40866)
Supplement: Multimedia Appendix 8 [file publichealth_v8i11e40866_app8.docx]

**Multimedia Appendix 8.** Surveillance metrics for domestic cluster infections using imported cases in New Zealand.

Multimedia Appendix 6 depicts the epidemic curve based on the empirical data from New Zealand. There was only one main outbreak of domestic cases between March 29 and April 5, 2020, which was followed by the peak of imported cases between March 22 and March 29, 2020.

Multimedia Appendix 5 shows the association between domestic cases lagging one-week behind imported cases in New Zealand (DIC=1687.9), which is smaller than the model with concurrent-week imported cases (DIC=2173.2) and those lagging two-week behind imported cases (DIC=2395.4) (data not shown but available upon requests). It shows that an increase in one imported case would increase the risk of domestic cluster infection per one imported case by 9.38% (95% CI 8.88% to 9.86%).

Although the scale of case number in Y axis in Multimedia Appendix 7 for New Zealand is remarkably higher than that of Figure 1 in Taiwan, patterns of both Taiwanese and New Zealand curves are similar. One exception is that there was one main outbreak of domestic cases (March 29 to April 5, 2020) followed by the peak of imported cases (March 22 to March 29, 2020) in New Zealand. Had relevant containment and mitigation measures been taken, as predicted by our proposed surveillance model in the week prior to the cluster infection, such a large-scale community-acquired outbreak might have been averted. However, it can be clearly seen that the subsequent cluster infection between April 11 and April 14, 2020 did not lead to large community-acquired infections in New Zealand, as similarly noted in Taiwan due to the adoption of containment and mitigation measures before cluster infection.

As far as the results of external prediction are concerned, there were three weeks between August 9 and August 29, 2020 in which the observed numbers of domestic cases were beyond the upper limit of surveillance level. The community outbreak in Auckland contributed the majority of this episode. The immediate response to take the measure of lockdown in Auckland successfully curbed the further spread of Covid-19.
